# Partial upper sternotomy for resection of a large substernal goiter in a high-risk patient

**DOI:** 10.1093/jscr/rjaf1085

**Published:** 2026-01-20

**Authors:** Nasim Kasiri, Alexander Pohlman, Andrea M Ziegler, Zaid M Abdelsattar, Julia M Coughlin

**Affiliations:** Stritch School of Medicine, Loyola University Chicago, 2160 S 1st Ave, Maywood, IL 60153, United States; Stritch School of Medicine, Loyola University Chicago, 2160 S 1st Ave, Maywood, IL 60153, United States; Department of Thoracic and Cardiovascular Surgery, Loyola University Medical Center, 2160 S 1st Ave, Maywood, IL 60153, United States; Department of Surgery, University of Illinois Chicago, 840 S Wood St., Chicago, IL 60612, United States; Stritch School of Medicine, Loyola University Chicago, 2160 S 1st Ave, Maywood, IL 60153, United States; Department of Otolaryngology, Loyola University Medical Center, 2160 S 1st Ave, Maywood, IL 60153, United States; Stritch School of Medicine, Loyola University Chicago, 2160 S 1st Ave, Maywood, IL 60153, United States; Department of Thoracic and Cardiovascular Surgery, Loyola University Medical Center, 2160 S 1st Ave, Maywood, IL 60153, United States; Department of Surgery, Edward Hines Jr. Veterans Affairs Hospital, 5000 5th Ave, Hines, IL 60141, United States; Stritch School of Medicine, Loyola University Chicago, 2160 S 1st Ave, Maywood, IL 60153, United States; Department of Thoracic and Cardiovascular Surgery, Loyola University Medical Center, 2160 S 1st Ave, Maywood, IL 60153, United States

**Keywords:** hyperthyroidism, partial sternotomy, substernal goiter

## Abstract

Substernal goiters (SGs) with significant mediastinal extension can present complex surgical dilemmas. While most SGs can be resected through a low cervical collar incision, a full median sternotomy may be required in select cases. Morbidly obese patients are susceptible to adverse outcomes following full sternotomy, such as wound infection and sternal dehiscence. Partial upper sternotomy is a safe, effective alternative that offers sufficient access to the mediastinum while minimizing surgical morbidity, making it a valuable approach in this high-risk population. We present the case of a 33-year-old female with a BMI of 47 kg/m^2^ who was found to have a large symptomatic SG with significant mediastinal extension. Due to the depth of extension, a combined cervical and trans-sternal approach was planned. A partial upper sternotomy was performed to avoid the morbidity of full sternotomy. The patient tolerated the procedure well without complications and was discharged home on postoperative day two.

## Introduction

A substernal goiter (SG) is defined as having >50% of its volume located below the sternal notch [1, 2]. Mediastinal extension is relatively uncommon, occurring in <5% of patients undergoing thyroidectomy [3]. Surgical resection remains the standard treatment for symptomatic SG with total thyroidectomy preferred to minimize recurrence. In 90%–95% of cases, this can be accomplished via a cervical approach [4]. However, in select cases, a median sternotomy may be necessary to access the intrathoracic portion of the mass, facilitating safer dissection while reducing the risk of recurrent laryngeal nerve (RLN) injury [5, 6].

While full median sternotomy provides broad exposure, it carries increased risks of wound complications, postoperative pain, and prolonged recovery: risks that are especially pronounced in patients with morbid obesity [5–7]. In contrast, partial upper sternotomy offers sufficient access to the mediastinum while minimizing surgical morbidity, making it a valuable approach in this high-risk population. To illustrate key management principles, we present the case of a 33-year-old morbidly obese woman with a symptomatic SG extending into the anterior mediastinum, which was successfully managed with total thyroidectomy via a low cervical collar incision and partial upper sternotomy.

## Case Report

**Figure 1:**
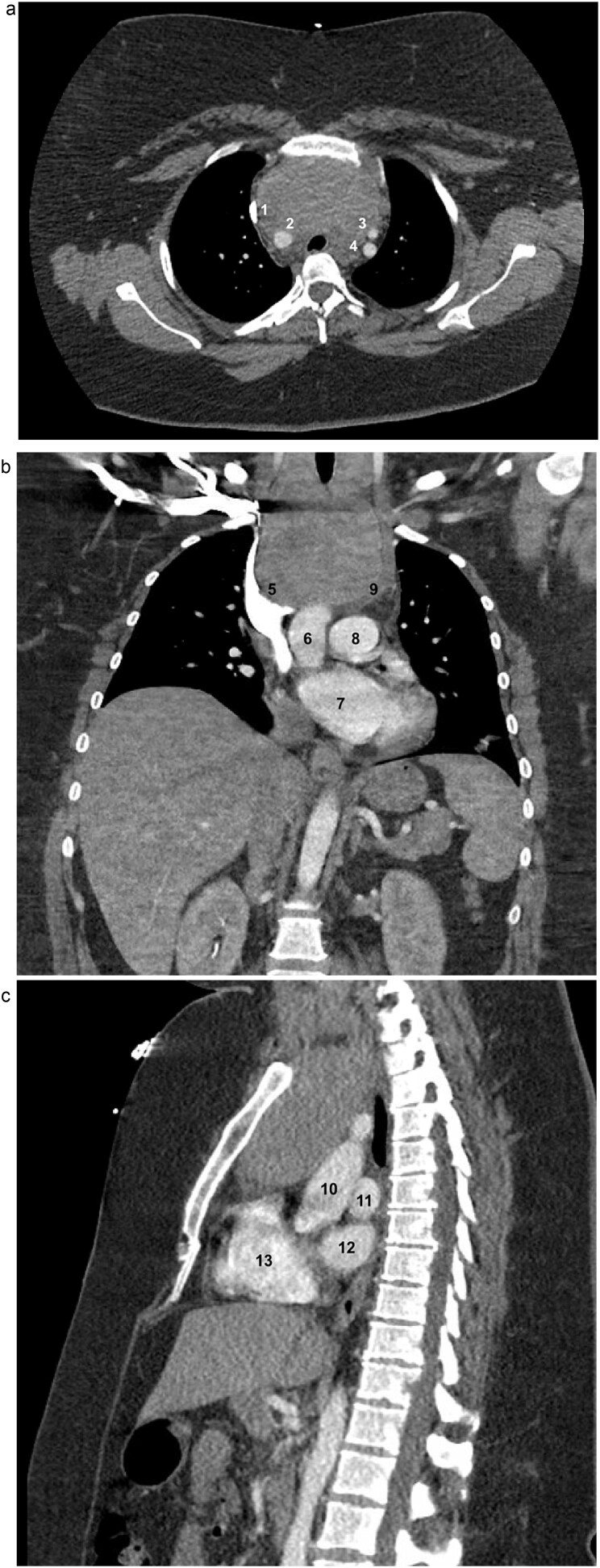
Axial (a), coronal (b), and sagittal (c) contrast-enhanced CT images of the chest demonstrating marked thyromegaly with retrosternal and intrathoracic extension (craniocaudal dimension up to 14 cm), resulting in tracheal narrowing at the thoracic inlet. In the axial view, the (1) superior vena cava (SVC), (2) brachiocephalic artery, (3) left common carotid artery, and (4) left subclavian artery are identified. In the coronal view, the (5) SVC, (6) ascending aorta, (7) left atrium (LA), (8) main pulmonary artery, and (9) brachiocephalic vein are identified. In the sagittal view, the (10) aorta, (11) left pulmonary artery, (12) LA, and (13) right ventricle are identified.

A 33-year-old woman with a history of morbid obesity (BMI = 47 kg/m^2^), gestational hypertension, and preeclampsia was incidentally found to have a large cervical mass identified during workup for fevers. Computed tomography (CT) scan revealed a 9.1 × 5.7 × 10.4 cm thyroid mass with extensive substernal extension and tracheal compression (Fig. 1a–c). Her family history was notable for thyroid cancer in her father and two paternal aunts. Due to the size of the mass, associated compressive symptoms, and strong family history of malignancy, a total thyroidectomy was recommended using a combined cervical and trans-sternal approach. Fine-needle aspiration demonstrated benign findings consistent with follicular colloid tissue. Given her body habitus and elevated risk of wound complications and sternal dehiscence, the sternal extension was limited to a partial upper sternotomy.

The patient was positioned supine with a shoulder roll placed to allow for maximal neck extension. The anterior neck and entire chest were prepped and draped below the xiphoid process in the event that a full median sternotomy would be required. A low cervical collar incision was made one fingerbreadth above the sternal notch along a natural skin crease. Subplatysmal flaps were raised superiorly to the thyroid cartilage and inferiorly to the sternal notch. The median raphe of the strap muscles was identified, it was divided longitudinally, and the strap muscles were retracted laterally to expose the thyroid gland. The superior poles of both the right and left thyroid lobes were dissected and ligated. Mobilization of the gland’s inferior portion was limited by dense adhesions extending into the mediastinum.

**Figure 2:**
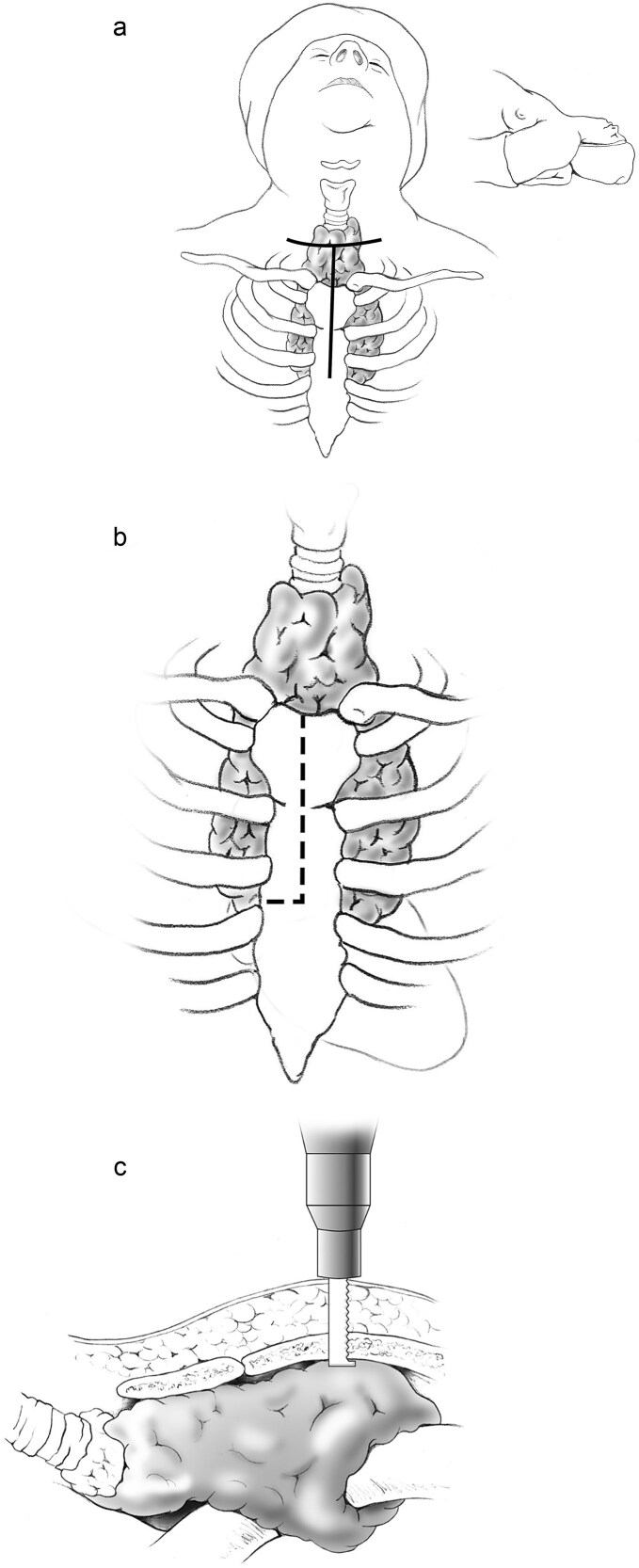
(a) A standard low cervical collar incision is created one fingerbreadth above the sternal notch, following a natural skin crease, with inferior extension from its midpoint to the angle of Louis. (b) A partial upper sternotomy is performed to the level of the third intercostal space, with transverse extension into the right third intercostal space. (c) The upper sternotomy is carried out using a standard sternal saw, and the transverse extension into the right third intercostal space is completed with an oscillating saw.

The skin incision was extended inferiorly from its midpoint to the Angle of Louis and carried down to the sternum (Fig. 2a). A partial upper sternotomy was performed to the level of the third intercostal space using a standard sternal saw, and a transverse extension into the right third intercostal space was created with an oscillating saw (Fig. 2b–c). An Estech retractor was used to carefully spread the sternal edges. The SG was meticulously dissected free from the underlying vascular structures and trachea and removed en-bloc with the thyroid gland. Intraoperative nerve monitoring was used throughout the procedure to confirm the preservation of both RLNs.

**Figure 3:**
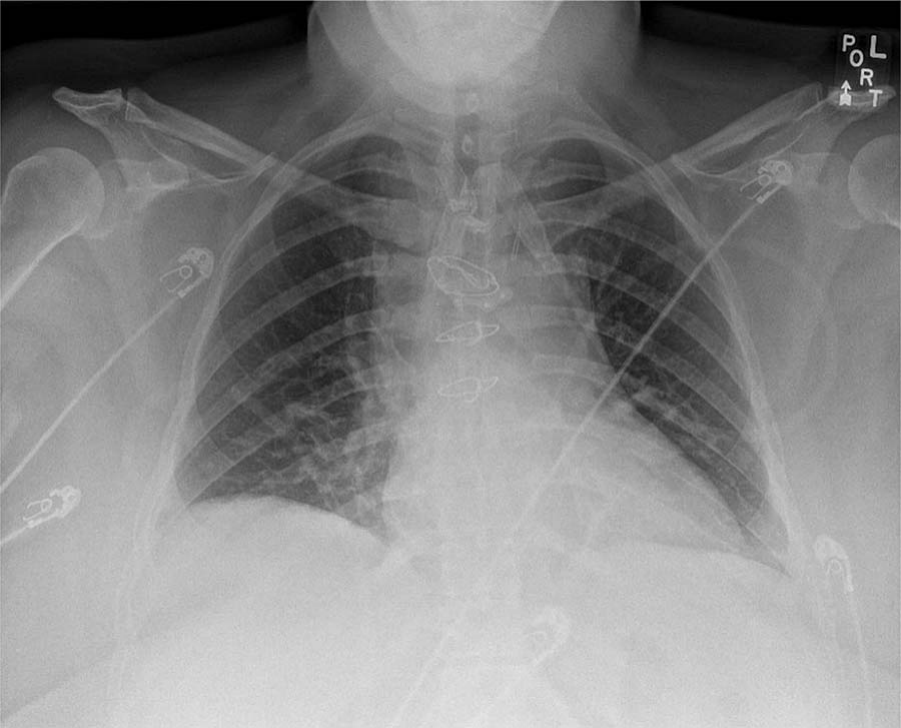
Postoperative chest X-ray demonstrating sternal wire and sternal plate closure.

A Jackson–Pratt drain was placed in the anterior mediastinum and exteriorized through a separate cervical stab incision. The sternotomy was closed using three standard single sternal wires and one sternal plate (Fig. 3). The strap muscles were re-approximated at midline, and the T-shaped incision was closed in layers with absorbable sutures. The patient had an uncomplicated postoperative recovery and was discharged home on postoperative day two. Final pathology confirmed a benign goiter with follicular nodular disease.

## Discussion

SGs are thyroid enlargements that extend into the mediastinum and often remain asymptomatic until significant intrathoracic growth leads to compressive symptoms. Patients may experience dyspnea, orthopnea, dysphagia, or chest pressure due to compression of the trachea, esophagus, or major vascular structures [8]. Surgical resection is generally recommended even for asymptomatic SGs, given the risk of acute airway compromise and potential malignancy [9].

A low cervical collar incision is sufficient in approximately 85%–95% of SG cases, but certain factors may necessitate a full sternotomy. Preoperative CT features such as extension >5 cm below the sternal notch, extension below the aortic arch, posterior mediastinal involvement, or limited cervical mobility of the goiter are predictive of the need for full sternotomy [10]. One study found that SGs with mediastinal extension of ≥5 cm beyond the sternal notch demonstrated a sensitivity of 94% and specificity of 86.5% for predicting the need for sternotomy [10]. A CT-based classification system showed that goiters extending to or below the aortic arch were associated with significantly higher rates of extra-cervical surgical access, aiding in preoperative planning and improving surgical outcomes [11]. Additionally, intraoperative findings such as dense adhesions or a history of prior neck surgery may also prompt conversion to sternotomy when cervical mobilization is deemed unsafe [12].

Obesity has been linked to an increased occurrence of deep sternal wound infection after surgery, as well as other complications including sternal dehiscence, prolonged ventilation, and prolonged hospital stay [13, 14]. Although partial upper sternotomy is more commonly employed in cardiac surgery, its application in the resection of SGs may offer significant advantages, particularly in high-risk patients with morbid obesity [15].

While literature on its use in thyroid surgery remains limited, our experience suggests that partial upper sternotomy can provide adequate exposure for safe resection of large SGs with significant extension posteriorly or inferiorly below the aortic arch. For obese patients, this less invasive approach may reduce operative morbidity, shorten hospital stays, and lower the risk of postoperative complications compared to full sternotomy [13–15]. As the burden of obesity continues to rise, partial upper sternotomy represents a promising and safer alternative for managing complex SGs in this high-risk population.
